# Early infections are associated with increased risk for celiac disease: an incident case-referent study

**DOI:** 10.1186/1471-2431-12-194

**Published:** 2012-12-19

**Authors:** Anna Myléus, Olle Hernell, Leif Gothefors, Marie-Louise Hammarström, Lars-Åke Persson, Hans Stenlund, Anneli Ivarsson

**Affiliations:** 1 Epidemiology and Global Health, Department of Public Health and Clinical Medicine, Umeå University, Umeå, Sweden; 2 Pediatrics, Department of Clinical Sciences, Umeå University, Umeå, Sweden; 3 Immunology, Department of Clinical Microbiology, Umeå University, Umeå, Sweden; 4 International Maternal and Child Health, Department of Women's and Children's Health, Uppsala University, Uppsala, Sweden

**Keywords:** Celiac disease, Epidemiology, Gluten amount, Infections, Infants and children

## Abstract

**Background:**

Celiac disease is defined as a ‘chronic small intestinal immune-mediated enteropathy precipitated by exposure to dietary gluten in genetically predisposed individuals’. Sweden has experienced an “epidemic” of celiac disease in children below two years of age. Celiac disease etiology is considered multifactorial; however, little is known regarding potential risk- or protecting factors. We present data on the possible association between early infectious episodes and celiac disease, including their possible contribution to the Swedish celiac disease epidemic.

**Methods:**

A population-based incident case-referent study (475 cases, 950 referents) with exposure information obtained via a questionnaire (including family characteristics, infant feeding, and the child’s general health) was performed. Celiac disease cases were diagnosed before two years of age, fulfilling the diagnostic criteria of the European Society for Pediatric Gastroenterology, Hepatology and Nutrition. Referents were randomly selected from the national population register after fulfilling matching criteria. The final analyses included 954 children, 373 (79%) cases and 581 (61%) referents, with complete information on main variables of interest in a matched set of one case with one or two referents.

**Results:**

Having three or more parental-reported infectious episodes, regardless of type of infection, during the first six months of life was associated with a significantly increased risk for later celiac disease, and this remained after adjusting for infant feeding and socioeconomic status (odds ratio [OR] 1.5; 95% confidence interval [CI], 1.1-2.0; P=0.014). The celiac disease risk increased synergistically if, in addition to having several infectious episodes, infants were introduced to dietary gluten in large amounts, compared to small or medium amounts, after breastfeeding was discontinued (OR 5.6; 95% CI, 3.1-10; P<0.001).

**Conclusion:**

This study suggests that having repeated infectious episodes early in life increases the risk for later celiac disease. In addition, we found a synergistic effect between early infections and daily amount of gluten intake, more pronounced among infants for whom breastfeeding had been discontinued prior to gluten introduction. Regarding contribution to the Swedish celiac disease epidemic, which partly was attributed to concurrent changes in infant feeding, early infections probably made a minor contribution via the synergistic effect with gluten amount.

## Background

Celiac disease is defined as a ‘chronic small intestinal immune-mediated enteropathy precipitated by exposure to dietary gluten in genetically predisposed individuals’ [1]. Approximately 1% of the population is affected; however, there are substantial differences regarding geographical distribution and changes over time [2-5]. A difference in screening-verified celiac disease prevalence between countries, as well as within Sweden, has been shown [5,6]. While a general increase over time in celiac disease occurrence has been recognized in several countries, a more complex pattern is apparent in Sweden. Between 1984 and 1996, celiac disease incidence displayed an epidemic pattern among children below two years of age consisting of a rapid four-fold increase in incidence rate followed by an equally abrupt decline one decade later [7,8]. The reasons behind the epidemic are still not fully understood, but approximately 45% of the cause was attributed to changes in infant feeding [9]. Screening of 12-year-olds born during the epidemic revealed a remarkably high celiac disease prevalence (3%) [6], emphasizing the need to further understand what caused the epidemic.

While differences in genetic predisposition of the population and gluten consumption could explain part of the differences in celiac disease occurrence [10], the complex and changing pattern of its occurrence indicates that environmental factors, besides gluten, affect celiac disease development. A seasonal difference in celiac disease diagnosis has been reported [11,12], indicating possible associations to environmental factors with seasonal occurrence, e.g. infections.

Previous studies have shown an association between celiac disease and factors related to infectious load, such as having siblings, attending child day care, and socioeconomic status, although these findings are still inconsistent [13-16]. Gastroenteritis increases gut permeability and subsequent gluten penetration, which could convey an increased risk for celiac disease [17]. Indeed, Stene et al. found an increased risk for celiac disease in children with repeated rotavirus infection measured by anti-rotaviral antibody positivity [14]. Later studies have not found an association between infections and celiac disease [18,19], and thus the evidence remains inconclusive.

We present data on the possible association between early infectious episodes and celiac disease, including their possible contribution to the Swedish celiac disease epidemic.

## Methods

### Study design and participants

**Figure 1:**
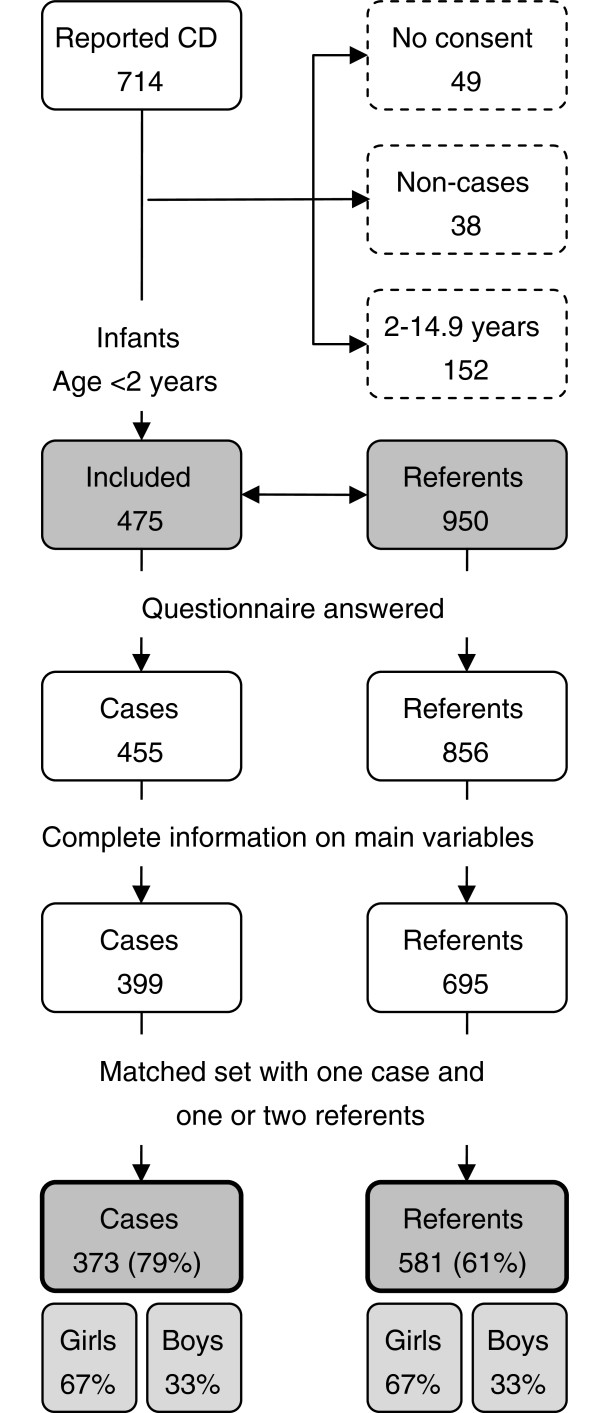
**Flow chart depicting participation in the study.** Numbers of children and percentages are given in the boxes. In total, 714 children with suspected celiac disease (abbreviated as CD in the figure) were reported to the register. Consent to participate was obtained for 475 cases below two years of age with a celiac disease diagnosis based on three consecutive small intestinal biopsies. Boxes with dotted lines indicate excluded children. Two referents to each case were randomly selected after matching criteria were fulfilled. Grey shaded areas indicate invited infants and those included in the final analysis.

We performed a population-based incident case-referent study based on a prospective incidence register entitled the Swedish National Childhood Celiac Disease Register [8,9]. Between 1992 and 1995, when the register encompassed 40% of the Swedish child population, all new reported cases were invited. Concurrently, two referents were randomly selected from the national population register [20] after fulfilling matching criteria (date of birth, sex and the family’s area of residence) [9]. In 475 infants (children below two years of age) celiac disease diagnosis was ascertained by three consecutive small intestinal biopsies, while on a normal diet, a gluten-free diet and after gluten challenge, respectively, according to the diagnostic criteria of the European Society for Pediatric Gastroenterology, Hepatology and Nutrition (Figure 1).

All families received a questionnaire entitled “Child Health in the 1990’s” where parents were asked about a multitude of events and exposures during the first years of their child’s life, including family characteristics, infant feeding, and the child’s general health, without revealing a special interest in celiac disease [9]. Inclusion criteria were defined as a matched set of one case with one or two referents with complete information on main variables of interest. The questionnaire was answered by 455 (96%) of the cases and 856 (90%) of the referents. The final analyses included 954 infants; 373 (79%) cases and 581 (61%) referents (Figure 1). All participating families gave informed consent. The study was approved by the Research Ethics Committees of all Swedish Me dical Faculties and the Swedish Data Inspection Board.

### Early infectious episodes

An *early* infectious episode was defined as occurring before six months of age, thereby maintaining a temporal relationship between infectious episodes (prior to) and celiac disease diagnosis. An infectious episode was defined as an infection from the panorama of childhood infections in Sweden *– common cold, otitis media*, *pneumonia, gastroenteritis, urinary tract infection, whooping-cough, scarlet fever, exanthema subitum (roseola infantum), or chicken-pox* – or an episode of *fever*, as this could be the only sign of viral infection in infants. The parents reported each infection as occurring once, twice or, three or more times. The infectious episodes were summarized and categorized into two categories, 0–2 episodes or ≥3 episodes. The cut-off (≥3) was set to the highest level where number of infectious episodes could be discriminated with certainty. The occurrence of gastroenteritis was additionally analysed separately. The cut-off was set at ≥1 episode, since few infants had experienced this infection during the first six months of life.

### Factors associated with infectious load

We investigated two additional factors for which previous studies have reported associations between both infectious load and celiac disease i.e. the presence of siblings and socioeconomic status [13-15]. Siblings included in the analyses were those of preschool age (below six years of age) at the time of birth of the infant participating in the study.

Socioeconomic status of the family was defined according to the Socio-Economic Index of Statistics Sweden, [21] which is based on the individual`s work position, taking into account the standard education level for each work position in the classification [20,21]. The Socio-Economic Index was categorized into two categories, high-medium and low [21].

In a post hoc approach we also investigated the relation between celiac disease and antibiotic treatment (including any kind of antibiotic) during the first six months of life.

### Infant feeding

As previously published in detail [9] and here described in overview, the duration of breastfeeding was defined as the period of time when the infant was exclusively or partially breastfed. Age at introduction of gluten was set to the first month of life during which flour from wheat, rye or barley was given. Breastfeeding status at the time of gluten introduction was categorized as discontinued before gluten introduction, continued the month of the gluten introduction, or continued beyond gluten introduction. The latter two were merged into one category for dichotomization. The amount of gluten-containing flour consumed per day, two weeks after the first portion, was calculated from the food frequency section of the questionnaire and standard recipes, and categorized into small-medium and large amounts with a cut-off at 16 grams/day [9].

### Statistical analyses

Analyses were based on 373 cases and 581 referents in matched sets, if not specified otherwise. We used conditional logistic regression to evaluate associations between categorical variables in both bivariate and multivariate analyses. Separate analyses for girls and boys were performed. Statistical interaction was analyzed on the additive scale. Proportion of cases attributable to exposure was estimated and decomposed into different exposures taking interaction into account. Data summarization and statistical analyses were done with PASW Statistics 19.0 (SPSS Inc, Chicago, IL). Statistical significance was defined as an odds ratio (OR) with 95% confidence interval (CI) not including 1.0, or a P-value <0.05.

## Results

### Study population characteristics

Among the 373 included cases, there was a predominance of girls (67%) (Figure 1). Median age at celiac disease diagnosis was 14 months (inter-quartile range 12–18 months) with a median age at symptom onset of 11 months (inter-quartile range 9–13 months). Data on age at symptom onset was missing for 5% of the cases.

We found no significant association between having siblings, compared to not having siblings, and celiac disease (OR 0.99; 95% CI, 0.74-1.3; P=0.92) (Table 1). Belonging to a family of lower socioeconomic strata, compared to high-medium strata, was associated with increased celiac disease risk (OR 1.5; 95% CI, 1.2-2.0; P=0.002) (Table 1). Adjusting for infant feeding and early infections reduced the risk estimate but it remained statistically significant (adjusted OR 1.3; 95% CI, 1.0-1.8; P=0.037). Breastfeeding at the time of introduction of gluten-containing foods into the infant`s diet was associated with decreased risk for celiac disease (Table 1). Introducing gluten in large amounts, compared to small and medium amounts increased the risk (Table 1). Age at gluten introduction was not an independent risk factor for celiac disease (adjusted OR 1.4; 95% CI, 0.9-2.4; P=0.16).

**Table 1:** Study population characteristics and association with celiac disease

	**Descriptive**			
**Exposures**^**a**^	**Cases**	**Referents**	**Bivariate analyses**	**P-value**
	**[n=373]**	**[n=581]**	**OR (95% CI) b**	
	**n (%)**	**n (%)**		
Siblings in preschool age				
Yes	215 (58)	333 (57)	1.0	
No	158 (42)	248 (43)	0.99 (0.74-1.3)	0.923
Family socioeconomic status				
High-Medium	193 (52)	358 (62)	1.0	
Low	180 (48)	223 (38)	1.5 (1.2-1.9)^c^	0.002
Breastfeeding at introduction of gluten^d^				
Discontinued	202 (54)	192 (33)	1.0	
Continued same month	88 (24)	147 (25)	0.55 (0.39-0.78)	0.001
Continued beyond	83 (22)	242 (42)	0.32 (0.23-0.45)	<0.001
Amount of flour per day two weeks after introduction^d^				
Small-Medium	189 (51)	373 (64)	1.0	
Large	184 (49)	208 (36)	1.9 (1.4-2.6)	<0.001

^a^ Exposures before six months of age.

^b^ Bivariate conditional logistic regression using matched sets of cases with 1–2 referents.

^c^ Preliminary version of this result has previously been included in book chapters [22-25].

^d^ These data has previously been published [9]. The data presented are based on the subset.

of infants included in the current study (see Methods) and is included in the multivariate analyses.

### Antibiotic treatment

Among the cases 26% (n=97) had been treated with any kind of antibiotic during the first six months of life. The corresponding proportion for the referents were 23% (n=134). No significantly increased risk for celiac disease was seen regarding antibiotic treatment (OR 1.2; 95% CI, 0.87-1.6; P=0.27). Separate analyses for girls and boys showed similar results (data not shown).

### Early infections and celiac disease risk

Having three or more parental-reported infectious episodes during the first six months of life was associated with a significantly increased risk for later celiac disease (OR 1.5; 95% CI, 1.1-2.0; P=0.009), as compared to fewer or no infectious episodes. Adjusting for infant feeding and socioeconomic status did not affect the risk estimate (Table 2). Furthermore, the results remained when excluding episodes of gastroenteritis. In total, 56 infants (29 cases and 27 referents) had experienced gastroenteritis during the first six months of life, as reported by the parents. Gastroenteritis (one or more episodes) was associated with increased risk for celiac disease (OR 1.8; 95% CI, 1.0-3.2; P=0.035), although the result did not reach statistical significance when adjusting for infant feeding and socioeconomic status (OR 1.8; 95% CI, 0.99-3.3; P=0.053) (Table 2). Separate analyses for girls and boys showed similar results (data not shown).

**Table 2:** Early infectious episodes and association with celiac disease

	**Descriptive**				
**Exposures**^**a**^	**Cases**	**Referents**	**Bivariate analyses**	**Multivariate analyses**	**P-value **^**d**^
	**[n=373]**	**[n=581]**	**OR (95% CI)**^**b**^	**OR (95% CI)**^**c**^	
	**n (%)**	**n (%)**			
Number of infectious episode(s)					
0-2	241 (65)	422 (73)	1.0	1.0	
≥3	132 (35)	159 (27)	1.5 (1.1-2.0)	1.5 (1.1-2.0) ^e^	0.014
Infectious episode(s) excluding gastroenteritis					
0-2	250 (67)	429 (74)	1.0	1.0	
≥3	123 (33)	152 (26)	1.5 (1.1-2.0)	1.4 (1.0-1.9)	0.035
Gastroenterits					
0	344 (92)	554 (95)	1.0	1.0	
≥1	29 (8)	27 (5)	1.8 (1.0-3.2)	1.8 (0.99-3.3)	0.053

^a^ Infectious episodes before six months of age.

^b^ Bivariate conditional logistic regression using matched sets of cases with 1–2 referents.

^c^ Multivariate conditional logistic regression using matched sets of cases with 1–2 referents, with each exposure adjusted separately for infant feeding and socioeconomic status (compare Table 1).

^d^ Corresponding P-value for the multivariate analyses.

^e^ Preliminary version of this result has previously been included in review articles/book chapters [22-27].

### Early infections and gluten amount

**Figure 2:**
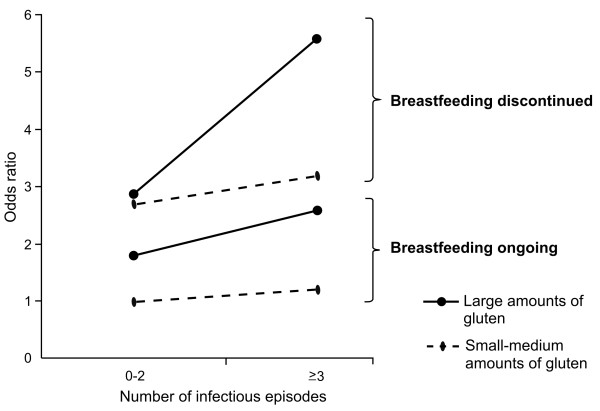
**Interaction between early infectious episodes and gluten amount.** Large amount of gluten-containing foods is represented by solid lines, and dotted line represents small-medium amounts. An interaction i.e. a synergistic effect, between infectious episodes and gluten is depicted by the non-parallelism between the two lower and two upper lines. The interaction was more pronounced in infants when breastfeeding was discontinued prior to gluten introduction. Odds ratios with 95% confidence intervals, with reference 0–2 early infections, gluten introduced in small-medium amounts during on-going breastfeeding, listed from lowest to highest risk estimate were; 1.2 (0.8-2.1), 1.8 (1.1-2.9), 2.6 (1.4-5.0), 2.7 (1.7-4.3), 2.9 (1.8-4.5), 3.2 (1.8-5.9) and 5.6 (3.1-10).

There was a statistical interaction between early infections and amount of gluten; i.e. the combined effect was higher than the sum of the effects, as depicted by the non-parallelism between the lower and upper two lines in Figure 2. The interaction was more pronounced among infants when breastfeeding had been discontinued prior to gluten introduction. The highest celiac disease risk (OR 5.6; 95% CI, 3.1-10; P<0.001) was seen if, in addition to several infectious episodes, these infants received gluten in large amounts, compared to small or medium amounts, two weeks after the introduction.

In this study, the proportion of cases attributable to exposure was in total 48%, whereof 4% of the effect was related to early infections alone, 38% to infant feeding alone, and 6% to the interactions between early infections, gluten amount and breastfeeding.

## Discussion

In this population-based incident case-referent study we found an increased celiac disease risk in infants with three or more parental-reported early infectious episodes (before 6 months of age), regardless of the type of infection. In addition, we found a synergistic effect between early infections and daily amount of gluten intake, which was more pronounced in infants for whom breastfeeding had been discontinued prior to gluten introduction.

In contrast to previous studies, we found no significant association between having siblings and celiac disease [13,14]. Unfortunately, we had no data on day care attendance, which could have confounded our findings. We found an increased risk for celiac disease in children from lower socioeconomic strata compared to medium-high strata. The risk decreased when adjusting for early infections and infant feeding, but it remained statistically significant, which implies that part, but not all of the risk can be attributed to these factors. Our findings are in agreement with previous observations in Swedish boys and individuals in the UK [15,28]. On the contrary, however, a recent large Swedish register-based study found a reverse association; a lower celiac disease risk in the lowest strata [16]. Similarly, Kondrashova et al. reported lower celiac disease prevalence in a Russian area than in a bordering area of Finland, suggesting that the lower socioeconomic status and/or inferior hygienic standard in Russia conveyed a protective effect against celiac disease [29]. Socioeconomic status is related to several underlying exposures that most likely differ both between countries and over time and thus further studies are needed to elucidate the role of these exposures and their combined effect on celiac disease risk.

The seasonal difference in celiac disease diagnosis [11,12] suggested that weaning during winter, when the infectious load is heavier, conveyed an increased celiac disease risk. Welander et al. investigated in a Swedish prospective cohort whether having an infection at the time of gluten introduction affected the risk for later celiac disease, but could not find any significant associations [19]. This could, however, be due to the fact that their study had the power to detect a 1.9-fold increase in celiac disease risk, and our findings suggest that the risk is lower (1.5-fold). As noted above, frequent rotavirus infections have been suggested to increase celiac disease risk [14] and rotavirus is a major cause of gastroenteritis in Swedish children [30]. However, in Sweden gastroenteritis is still relatively uncommon early in life [19]. In our study we could not find a significantly increased celiac disease risk due to parental-reported gastroenteritis, although this might be due to too few infants with this infection (Table 2). The finding of an increased celiac disease risk in infants with three or more non-gastrointestinal early infections suggests that infections, irrespective of localization, affect celiac disease risk, although the molecular mechanisms behind this finding remain to be clarified.

On the other hand, our finding that the celiac disease cases experienced more infections early in life could be related to an inherited genetic susceptibility for both celiac disease and infections. Previous studies have reported a moderately increased risk for a number of infections in celiac disease cases, for example hospital admission for influenza, although these findings could also be related to nutritional deficiency, increased mucosal permeability or other factors [31]. It is, however, reasonable to assume that an increased risk could be related to even earlier exposures than the ones investigated in the current study. Neonatal infections have been associated with increased celiac disease risk [13,32], although this observation was not replicated in another study [18]. Mårild et al. found an increased risk for celiac disease following elective, but not emergency, caesarean section [18]. The former affects the gut colonization and subsequently the microbiota in the infant, which could affect the risk for celiac disease, as differences between celiac disease cases and non-cases in microbiota composition have been shown [33-36].

The microbiota interacts with genetic and environmental factors such as infant feeding, through a complex immunological process resulting in establishment of oral tolerance to gluten, or failure to do so resulting in celiac disease [37]. Infections early in life, when the gut microbiota is developing, could possibly result in a different microbiota, affecting both intestinal immune responses and mucosal barrier function and thereby also the risk for celiac disease [38]. Further, antibiotic treatment affects the gut microbiota and might therefore be a risk factor for celiac disease [39]. We speculated that the increase in celiac disease occurrence during the past decades could be related to the increased use of antibiotics during the same time period. However, in our post hoc approach to antibiotic treatment and celiac disease we found no significant association, although this could be due to lack of data on type of antibiotics used. Similarly to antibiotics and early infections, early vaccinations also influence the immune system. We have previously shown that BCG vaccination is associated with a protective effect for celiac disease, although this has to be interpreted with caution [40]. Adjusting the association between early infections and celiac disease for BCG vaccination status did not affect the association (data not shown). Other vaccinations within the Swedish national program were not risk factors [40].

Concurrent with the beginning and end of the Swedish celiac disease epidemic, infant feeding changed [7]. As infant feeding affects the microbiota [37,38], these changes could have resulted in the increased frequency of rod-shaped bacteria seen in jejunal biopsies from both untreated and treated celiac disease cases born during the epidemic, as compared to celiac disease cases born afterwards and healthy controls [41,42]. Alternatively, the rod-shaped bacteria with celiac disease promoting characteristics constitute an independent phenomenon and part of the celiac disease epidemic may be attributed to them. Furthermore, although there are no available ecological data in Sweden to explore whether the infectious panorama changed during the epidemic period, the synergistic effect between early infections and gluten amount, to which approximately 6% of the cases could be attributed, implies that early infections reinforced the changes in gluten amounts consumed [7]. Thereby early infections probably had a minor contribution to the Swedish celiac disease epidemic, irrespective of changes in the infectious panorama.

A limitation of our study is that it was retrospective and based on parental reports in a questionnaire. Consequently, there was an inherent risk for recall bias, even if the interest in celiac disease was not indicated in the questionnaire and incident recruitment of both cases and referents was used. The infant feeding data has previously been evaluated using a “multiple imputation procedure” without effect on the results [9]. Our study was limited to symptomatic infectious episodes. Difficulties in correctly identifying infectious episodes in young infants were the same for parents of both cases and referents, although the lack of data on specific pathogen and severity of the infection still constitutes a limitation.

## Conclusions

This study suggests that having repeated infectious episodes early in life increases the risk for later celiac disease. In addition, we found a synergistic effect between early infections and daily amount of gluten intake, more pronounced among infants when breastfeeding had been discontinued prior to gluten introduction. This synergistic effect substantiates the importance of breastfeeding with respect to celiac disease risk, especially in a setting with high infectious load. Regarding a contribution to the Swedish celiac disease epidemic, which partly was attributed to concurrent changes in infant feeding, early infections probably made a minor contribution via the synergistic effect with the amount of gluten.

## Abbreviations

CI: Confidence interval; OR: Odds ratio.

## Competing interests

The authors declare that they have no competing interests.

## Authors’ contributions

AI, LÅP and OH were responsible for the original design and data collection. The first draft of the manuscript was prepared by AM who also performed the statistical analyses, with support from HS, LG and MLH contributed substantially with specific knowledge within their respective fields of knowledge. All authors participated in data interpretation, critical revision of the manuscript and approved its final version.

## Pre-publication history

The pre-publication history for this paper can be accessed here:

http://www.biomedcentral.com/1471-2431/12/194/prepub
